# Strains of Pathological Protein Aggregates in Neurodegenerative Diseases

**DOI:** 10.15190/d.2017.8

**Published:** 2017-09-30

**Authors:** Xinzhu Wang, Zeinab Noroozian, Madelaine Lynch, Nicholas Armstrong, Raphael Schneider, Mingzhe Liu, Farinaz Ghodrati, Ashley B. Zhang, Yoo Jeong Yang, Amanda C. Hall, Michael Solarski, Samuel A. Killackey, Joel C. Watts

**Affiliations:** Department of Laboratory Medicine and Pathobiology, University of Toronto, Toronto, ON, Canada; Tanz Centre for Research in Neurodegenerative Diseases, University of Toronto, Toronto, ON, Canada; Sunnybrook Research Institute - Biological Sciences, Toronto, ON, Canada; Department of Medicine, Division of Neurology, University of Toronto, Toronto, ON, Canada; Department of Pharmacology and Toxicology, University of Toronto, Toronto, ON, Canada; Department of Biochemistry, University of Toronto, Toronto, ON, Canada

**Keywords:** Prion, strain, Creutzfeldt-Jakob disease, Alzheimer’s disease, Parkinson’s disease, amyotrophic lateral sclerosis, Aβ, tau, α-synuclein, SOD1, yeast prion, structure, transmission, evolution

## Abstract

The presence of protein aggregates in the brain is a hallmark of neurodegenerative disorders such as Alzheimer’s disease (AD) and Parkinson’s disease (PD). Considerable evidence has revealed that the pathological protein aggregates in many neurodegenerative diseases are able to self-propagate, which may enable pathology to spread from cell-to-cell within the brain. This property is reminiscent of what occurs in prion diseases such as Creutzfeldt-Jakob disease. A widely recognized feature of prion disorders is the existence of distinct strains of prions, which are thought to represent unique protein aggregate structures. A number of recent studies have pointed to the existence of strains of protein aggregates in other, more common neurodegenerative illnesses such as AD, PD, and related disorders. In this review, we outline the pathobiology of prion strains and discuss how the concept of protein aggregate strains may help to explain the heterogeneity inherent to many human neurodegenerative disorders.

## SUMMARY

1. *Introduction to prions and prion strains*
2. *Methods for delineating prion strains*
3. *Strains of PrPSc in the human prion diseases*
4. *The structural biology of prion strains*
5. *Prion strain transmission, adaptation, and mutation*
6. *Strains of protein aggregates in other neurodegenerative diseases*
7. *Conclusions***

## 1. Introduction to prions and prion strains

### 1.1 Prion diseases and prion propagation

The transmissible spongiform encephalopathies (TSEs), also referred to as prion diseases, are invariably fatal neurodegenerative disorders that affect both animals and humans. Animal prion diseases include scrapie, which affects sheep and goats; bovine spongiform encephalopathy (BSE; also called mad cow disease), which affects cattle; transmissible mink encephalopathy (TME), which affects farmed mink; and chronic wasting disease (CWD), which is known to affect deer, elk, and moose. In addition to the classical versions of these disorders, it is now accepted that atypical forms exist, such as atypical scrapie and atypical BSE, which exhibit unique molecular and pathological properties^1^. Diverse prion diseases such as Creutzfeldt-Jakob disease (CJD) are also recognized in humans and can be categorized into three types: sporadic, hereditary, and acquired (infectious). Classical forms of scrapie, BSE, CWD, and TME are acquired prion disorders, whereas the emergent atypical forms may represent sporadic prion diseases of animals. Many other animal species including non-human primates and small laboratory animals have also been experimentally infected with TSEs from various origins.

A hallmark of prion disorders is that they can be transmitted within or between species under both natural and experimental conditions. The transmissible agent in these diseases consists of a small infectious protein that is resistant to processes that inactivate nucleic acids. In 1982, Stanley Prusiner coined the term “prion” to describe these proteinaceous infectious particles, and the protein-only hypothesis for prion disease was outlined^2^. It is now known that prions consist of a single protein: the prion protein (PrP). There are two main structural isoforms of PrP: PrP^C^ (cellular PrP) and PrP^Sc ^(PrP scrapie)^3^. PrP^C^, which is encoded by the *PRNP* gene in humans, is a monomeric and largely α-helical glycoprotein that is anchored to the outside of the cell membrane by means of a glycosylphosphatidylinositol (GPI) anchor. The normal function of PrP^C^ is still debated^4,5^, but it is known to be involved in myelin maintenance within the peripheral nervous system^6,7^, which is unlikely to be related to its role in the prion disorders. During prion disease, the pathological PrP isoform, PrP^Sc^, is generated from PrP^C^ via a conformational conversion mechanism that remains to be elucidated. Unlike PrP^C^, PrP^Sc^ is enriched in β-sheet content, insoluble, prone to forming aggregates, resistant to protease digestion, and neurotoxic^3^. The conversion of PrP^C^ into PrP^Sc^ is believed to be the central pathogenic event in the prion disorders, since mice lacking PrP^C^ expression are completely resistant to prion disease^8^.

PrP^Sc^ is a self-replicating or self-propagating protein: it is capable of recruiting PrP^C^ and catalyzing the misfolding of PrP^C^ into additional copies of PrP^Sc^, referred to as prion replication. The ability of PrP^Sc^ to self-propagate underlies the infectious nature of the prion disorders. Introduction of an exogenous source of PrP^Sc^, such as feed contaminated with BSE prions, into the body can trigger the conversion of host PrP^C^ into new PrP^Sc^ molecules. This is also the basis for the experimental transmission of prion disease to laboratory rodents. At the molecular level, a cascade of prion replication leads to the spreading of prion aggregates within the brain. The progressive accumulation and deposition of PrP^Sc^ aggregates within the central nervous system (CNS) leads to the pathological hallmarks of prion disease: neuronal degeneration and death, reactive astrocytic gliosis, and spongiform degeneration of the brain parenchyma. The most infectious and toxic prion particles are thought to be smaller, oligomeric aggregates of PrP^Sc^9,10^^, as they can readily spread between cells.

### 1.2 Prion strains

Prion strains are different types or isolates of prions that produce a characteristic phenotype. Prion strains were first recognized in 1961, when Pattison and Millson published their observations from experimentally produced scrapie in goats. Throughout their experiments, they observed two types of symptoms in goats inoculated with sheep scrapie: the nervous phenotype, which broadly included symptoms such as hyperexcitability and hypersensitivity, and the scratching phenotype, in which the goats had an increased tendency to excessively scratch themselves^11^. Not only were they able to obtain an inoculum that produced the same clinical symptoms as the original diseased donor animal, but these characteristic symptoms also persisted throughout multiple passages. Pattison and Millson suggested that these clinical symptoms are inherent characteristics of each inoculum “type”. This significant discovery showed that scrapie agents from the same source could lead to clinically different syndromes.

**Figure 1:**
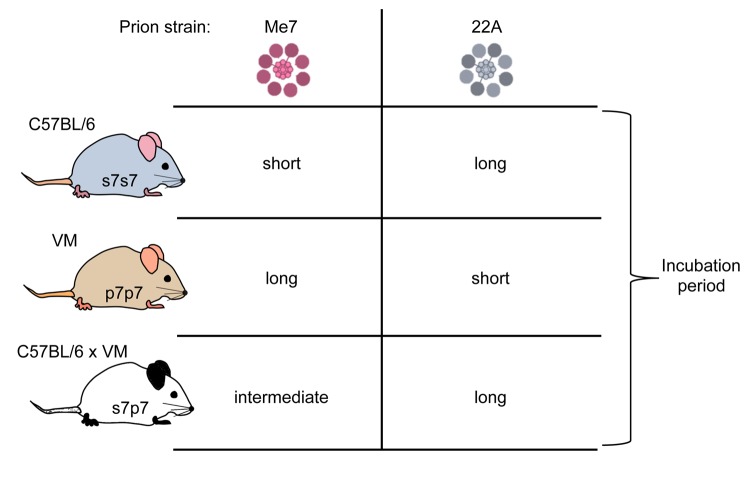
Evidence for the existence of prion strains from transmission studies using inbred mice The relative incubation periods for the prion strains Me7 and 22A depend on the sinc genotype of the mouse line^13^. C57BL/6 mice are homozygous for the s7 sinc allele whereas VM mice are homozygous for the p7 allele. The presence of distinct incubation periods for different prion isolates in the same line of inbred mice provided early evidence for the existence of prion strains.

During the early 1970’s, a number of major discoveries regarding prion strains were made. Among them were the identification of *sinc*, a gene in mice which controls the incubation period of the scrapie agent^12^, and that different scrapie agents can lead to distinct incubation periods in the same mammalian host even with the same *sinc* genotype^13^. These experiments utilized two prion strains, Me7 and 22A, in combination with two strains of inbred mice, C57BL/6 and VM, which were homozygous for the s7 allele and p7 allele of *sinc*, respectively. The Me7 prion agent had a shorter incubation time in C57BL/6 mice than in VM mice whereas the incubation lengths were reversed for the 22A strain (**Figure 1**). The results of this experiment strengthened the idea of prion strains having distinct characteristics and confirmed the involvement of the *sinc *gene in controlling responses to various prion agents. It was ultimately shown that the s7 and p7 alleles corresponded to allelic variants of PrP^14^ and that the *sinc* and *Prnp* genes were congruent^15^. In addition to independent incubation periods, the intensity and distribution of gray matter lesions were identified as a distinguishing feature of different strains of the scrapie agent^16^. These distinct properties among scrapie strains suggested the existence of differences at the molecular level and proposed new directions for prion research.

As the evidence for prion strains accumulated and observations of phenotypically distinct prion “agents” were made, the protein-only hypothesis^2^ was met with resistance. In bacteria and viruses, unique strains arise due to changes in the nucleic acid genomes of the agents, leading to the emergence of new properties. The notion that PrP^Sc^ propagates by converting endogenous PrP^C^ into prions by inducing a specific fold seemed to contradict the existence of prion strains. Instead, some argued that strain-specific properties of prions must be encoded by a nucleic acid genome^17,18^. However, no supporting evidence for the existence of a prion-specific nucleic acid was found, and instead, the advancement of molecular biology techniques confirmed biochemical differences between PrP^Sc^molecules, suggesting that strain phenotypes may be encoded within different conformations of PrP^Sc^19^^. For example, although scrapie-associated fibrils isolated from animals infected with different scrapie strains (Me7, 263K, and 139A) had a related class of structures, the individual strains exhibited distinguishable morphologies, sedimentation rates, protein compositions and sensitivity to proteinase K (PK) digestion^20^. Additionally, purified PrP^Sc^molecules from two strains of hamster-adapted TME prions, “hyper” (HY) and “drowsy” (DY), displayed different biochemical characteristics^21^. Similar molecular differences, which were maintained upon transmission to mice, were observed with human prion strains^22^. Moreover, eight different strains of hamster prions could be differentiated based on the relative exposure of antibody epitopes in PrP^Sc^23^^. Collectively, these studies have provided evidence beyond a reasonable doubt that the molecular properties of prion strains are encoded by unique conformations of PrP^Sc^ aggregates. With this knowledge, prion strains can be defined as conformational variants of PrP^Sc^ aggregates with distinct biochemical and molecular traits that can produce distinct clinical and/or neuropathological manifestations.

## 2. Methods for Classifying Prion Strains

With the discovery of prion strains, the development of methods to differentiate between and classify them became necessary. Fortunately, the early evidence for the existence of prion strains also provided insight into how these species could be classified. As mentioned previously, seminal work demonstrated that prion strains produce distinct clinical phenotypes in experimental animals, exhibit unique incubation periods, and cause different distributions and intensities of gray matter lesions in diseased animals^11,16,21,24^. More recently, with the discovery of PrP and advances in laboratory technology, more sensitive biochemical assays have been developed. The correct classification of prion strains is critical for understanding the aetiology of prion diseases in humans and animals, since the clinical course and potential for transmissibility may depend on the specific strain. In general, the techniques for classifying prion strains can be grouped into three categories: clinical, neuropathological, and biochemical.

### 2.1 Clinical Methods for Classifying Prion Strains

One of the first methods used to differentiate between prion strains came from early observations that two distinct clinical phenotypes can exist in goats afflicted with scrapie (nervous and scratching)^11^. A similar phenomenon of clinical variation was observed following the transmission of TME prions to Syrian golden hamsters, in which two distinct syndromes developed after three passages (HY and DY)^21,24^. In the HY syndrome animals displayed symptoms of hyperesthesia and ataxia, while in the DY syndrome hamsters were characterized by lethargy, slowed movements, and lack of coordination. While classification based on clinical presentation is a relatively crude method to differentiate prion strains in experimentally infected animals, it is one of the few *in vivo* methods that currently exist.

**Figure 2:**
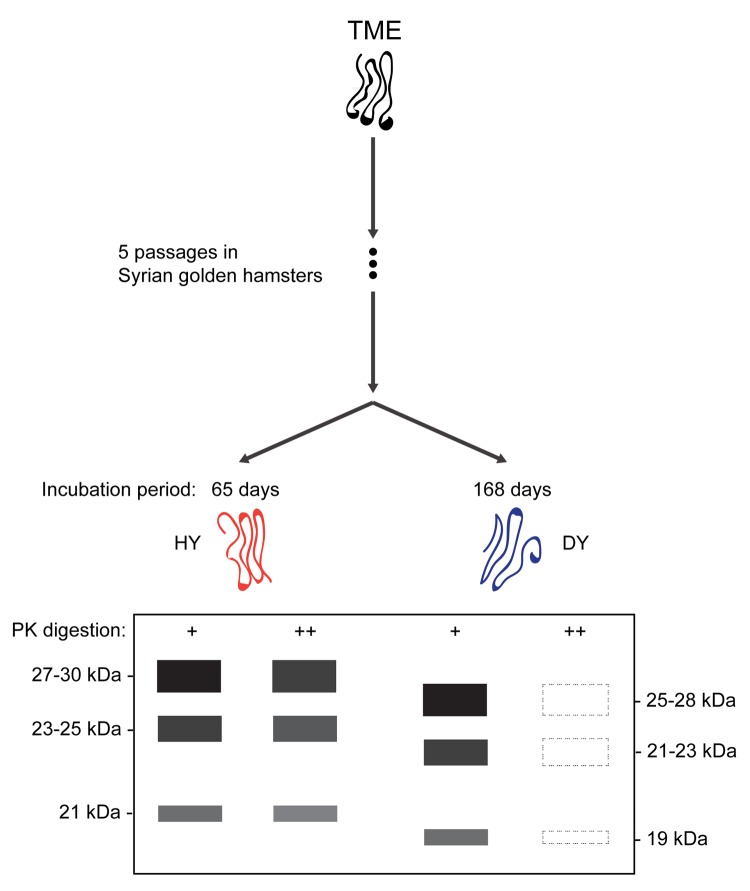
Generation and characterization of the HY and DY prion strains Repeated passaging of the Stetsonville transmissible mink encephalopathy (TME) isolate in Syrian golden hamsters led to the emerge of the HY and DY strains, which exhibited incubation periods of 65 or 168 days, respectively^24^. A representative PrP immunoblot of HY (left) and DY (right) strains following limited PK digestion (+) is also shown. Note the difference in electrophoretic mobility and sensitivity to extended PK digestion (++) between the two strains (represented by band shading). HY and DY strains have a similar ratio of the three PrP^Sc^ glycoforms.

Another difference between certain prion strains is their strikingly different incubation periods upon inoculation into rodents. If all experimental variables (such as prion titre) are kept constant, the time between infection with PrP^Sc^ and the onset of symptoms is stable for a given strain. In the case of hamster prions, the HY and DY TME strains have incubation periods of 65 and 168 days, respectively, despite the fact that these strains were derived from the same source (**Figure 2**)^24^. Similarly, in C57BL/6 mice that express the PrP-A allele, the commonly used RML strain of mouse-adapted scrapie prions has an incubation period of ~150 days, whereas the BSE-derived 301V strain has an incubation period of ~270 days^25^. However, the length of the incubation period is also dependent on the specific PrP allele expressed by the host. For example, in mice that express the PrP-B allele, the relative incubation lengths are reversed for the RML and 301V strains^26,27^.

### 2.2 Neuropathological Methods for Classifying Prion Strains

Examination of the brains of prion-infected animals has revealed that different strains cause distinct patterns of neuropathology, a characteristic that can be used as a criterion for strain identification. The most common microscopic change observed in prion disease is neuronal vacuolation and spongiform degeneration of the neuropil within the gray matter^16^. The intensity of neuronal vacuolation and spongiform change can be scored in various regions of the brain to provide a “lesion profile” for a given strain, which is highly characteristic and stable upon serial passage^16,28^. In addition, the distribution and morphology of PrP^Sc^ deposits can be unique to each prion strain. Immunohistochemistry (IHC) analysis using PrP antibodies reveals that PrP^Sc^ deposits are confined to areas of degeneration in some strains, while more widespread in others^29^. The morphology of these deposits provides another criterion for strain discrimination. Some strains form diffuse, non-fibrillar PrP^Sc^ aggregates, while others produce dense, congophilic, fibrillar plaques^30^. Overall, microscopic examination of PrP^Sc^-infected brain tissue is a powerful method for strain discrimination and is often accurate enough to correctly identify strains in the absence of other methods.

More recently, luminescent conjugated polymers (LCPs) have been applied to the study of prion strains. LCPs are molecular probes that exhibit conformation-dependent emission spectra when bound to protein aggregates. LCPs were capable of discriminating between four immunohistochemically indistinguishable prion strains^31^, suggesting that LCPs can be used in conjunction with classical neuropathological techniques as a sensitive method for strain identification.

### 2.3 Biochemical and Cellular Methods for Classifying Prion Strains

The discovery of PrP allowed for the development of more sensitive methods for the classification of prion strains based on their biochemical and molecular properties. Immunoblotting of PrP^Sc^ following limited digestion with PK reveals a number of these properties. Firstly, the electrophoretic mobility of various strains may differ due to the differential exposure of PK cleavage sites, which gives rise to PK-resistant PrP fragments of variable size. For example, following PK digestion, the unglycosylated PrP band of the HY TME strain has an electrophoretic mobility of 21 kDa, whereas the unglycosylated PrP band of the DY strain has an electrophoretic mobility of 19 kDa^21^ (**Figure 2**). A similar difference is seen between the Type 1 and 2 strains of sporadic CJD (sCJD), which have electrophoretic mobilities of 21 and 19 kDa, respectively^32^. Prion strains are also easily classified on Western blot based on the relative proportions of the three PK-resistant PrP glycoforms^33-35^. Large-scale analyses of human CJD cases revealed that the glycoform ratios vary between sCJD and variant CJD (vCJD) cases, with the monoglycosylated band predominating in sCJD cases and the diglycosylated band being the most abundant in vCJD cases^33^. The fact that PrP^Sc^ glycoform abundance can be strain-specific provided early evidence for the link between vCJD and BSE^36^. Further classification is based on a strain’s relative sensitivity to PK digestion^21^. Digestion of PrP^Sc^ with higher PK concentrations revealed that some strains have a stronger resistance to degradation than others^20,37^ (**Figure 2**). These characteristics of PrP^Sc^ (electrophoretic mobility, glycosylation pattern, and PK resistance) are three of the main biochemical properties used to classify prion strains.

Prion strains can also be differentiated according to the sedimentation properties of PrP^Sc^. During prion extraction, PrP^Sc^ from hamster brains infected with the HY strain was distributed in different fractions than PrP^Sc^ from brains infected with the DY strain^21^, suggestive of a difference in solubility between the two strains. Further work using sedimentation velocity techniques revealed that, in most strains, PrP^Sc^ is highly concentrated in the middle of an iodixanol gradient^38^. However, in some strains, the most infectious particles sediment very slowly and are found in higher fraction numbers. Slowly sedimenting particles, which represent smaller aggregates, may be a feature of strains that are able to produce a more aggressive disease. Additionally, prion strains appear to form different types of aggregates with strain-specific density distributions^37^.

The relative conformational stabilities of prion strains have also been used as a criterion for strain discrimination. When exposed to increasing concentrations of guanidine hydrochloride (GdnHCl), PrP^Sc^ gradually becomes denatured^39^. The concentration of GdnHCl required to denature PrP^Sc^ is strain-specific, with each strain exhibiting a characteristic half-maximal denaturation concentration value. It has been found that prion strains with lower conformational stabilities propagate faster than strains with higher stabilities^40^, although this may not be true for all strains^41^. Prion strains can also display differences in solubilization temperature^30^ and can differ with respect to the rate of inactivation with increasing temperature^42^.

In the past two decades, more modern biochemical techniques have been developed for the study of prion strains. One such method is the conformation-dependent immunoassay (CDI)^23^. This technique differentiates between strains by measuring the amount of PrP-directed antibody binding before and after denaturation, given that some PrP epitopes are variably buried in different strains of PrP^Sc^. The CDI has been shown to be effective in distinguishing between eight strains that possess similar incubation periods and Western blot profiles^23^. Real-time quaking-induced conversion (RT-QuIC) is a technique originally developed for the sensitive detection of PrP^Sc^ in biological samples^43^. In this assay, PrP^Sc^ seeds induce the formation of Thioflavin T-reactive amyloid fibrils from recombinant PrP substrates. RT-QuIC is able to distinguish between various strains of BSE and scrapie based on their relative ability to seed fibril formation when employing a variety of recombinant PrP substrates^44-46^.

The relative ability of specific prion strains to infect different cell lines has also been used for strain discrimination. Using a panel of four cell lines (PK1, CAD5, LD9, and N2a-R33), it was demonstrated that prion infectivity depends on the strain and cell line used^47^. For example, the 22L strain could be propagated in all four cell lines, while the 301C strain was only propagated in CAD5 cells, which were susceptible to all strains studied. These findings suggest that strains have an intrinsic “virulence” associated with them and that different cell lines have a varying susceptibility to infection^47^.

As the list of methods used to classify prion strains continues to grow, so does our understanding of the fundamental basis of prion strain diversity. While other methods exist, such as those based on differences in immunoreactivity^21^, prion titre in brains^21^, and binding affinity for copper^35^, the ones discussed above represent the main methods currently in use. It is evident, however, that no single method alone is sufficient for strain classification, given that some strains can be similar in one aspect (e.g. incubation time), but different in another (e.g. protein conformation)^23^. In the future, it is likely that prion strain classification systems will move away from crude methods, such as clinical phenotype and lesion profile, to more advanced biochemical methods that can detect subtle differences between strains at the molecular level. An ideal classification method would be rapid and highly sensitive, such that it could be used in a clinical setting to identify the pathogenic strain in a patient with prion disease. This information would inform a physician about their patient’s clinical course and allow them to choose the most suitable treatment.

## 3. Strains of PrPSc in the Human Prion Diseases

The most common human prion disorder is sCJD, which accounts for ~85% of all cases. Another sporadic prion disorder is the recently described variably protease sensitive prionopathy (VPSPr)^48^. Familial prion diseases account for 10 to 15% of cases and include familial CJD, Gerstmann-Sträussler-Scheinker disease (GSS), and fatal familial insomnia (FFI). All familial prion diseases reported to date are caused by mutations in the *PRNP* gene^32^. Alternatively, iatrogenic CJD, variant CJD, and Kuru are acquired prion diseases. Iatrogenic CJD has been reported after neurosurgical procedures, such as cerebral electrode implantation, corneal and dura mater transplants, and human growth hormone therapy, presumably through transmission of PrP^Sc^ between humans^49^. A causal link between consumption of BSE-contaminated beef and vCJD has been substantiated by data from experimental prion transmissions^33,50^. Unlike the other prion diseases, Kuru has been observed exclusively in the Fore tribe of Papua New Guinea, and transmission appears to occur via ritualistic cannibalism^51^.

The wide spectrum of human prion disorders, all of which result from the misfolding of PrP, can be at least in part explained by the existence of unique strains of human PrP^Sc^. As with the animal prion diseases described above, human prion diseases can also present with varying clinical symptoms. For example, classical sCJD is a rapidly progressive dementia with onset between the ages of about 40 and 90 years and death within weeks to a few years following diagnosis^32,52^. Less common versions of the disease include the Heidenhain variant, which is a form of sCJD with visual symptoms and severe occipital pathology^53^, and a variant with prominent ataxia and cerebellar pathology^54^. The clinical presentations of GSS and FFI are quite different from that of sCJD: GSS is predominantly a cerebellar syndrome characterized by progressive ataxia, whereas FFI patients initially present with sleep abnormalities and hallucinations^55^. Moreover, the recent demonstration of prions in patients with diarrhea and autonomic neuropathy has further expanded the clinical spectrum of the prion disorders^56^. Rates of disease progression can also vary amongst the human prion diseases. For instance, patients with vCJD are generally younger at disease onset and survive longer than patients with sCJD.

### 3.1 Molecular Classification of Sporadic CJD

**Figure 3:**
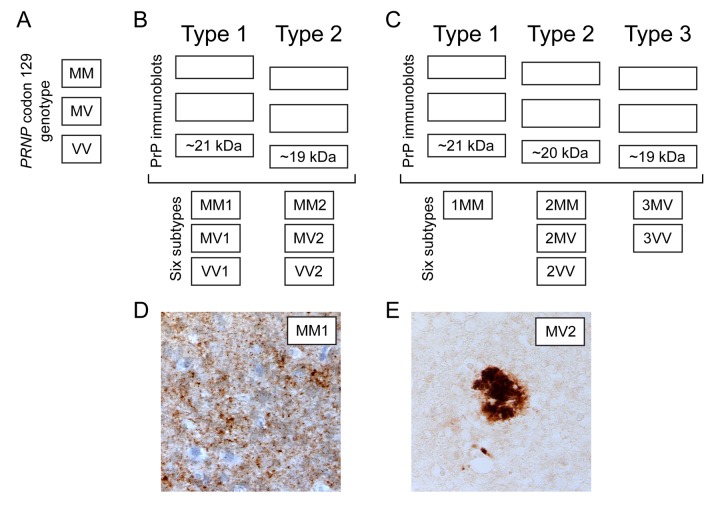
Strains of PrPSc in sporadic CJD patients **(A**) The three possible codon 129 *PRNP* genotypes. (**B**) Classification of sCJD strains according to the “Gambetti” system. Immunoblot profile showing Type 1 (~21 kDa) and Type 2 (~19 kDa) PrP^Sc^ as defined by the size of the PK-resistant PrP^Sc^ fragments. In this system, six subtypes of sCJD are defined according to the PrP^Sc^ type and the codon 129 genotype: MM1, MV1, VV1, MM2, MV2, and VV2. (**C**) Classification of sCJD strains according to the “London” system. In this system, three distinct sizes of PK-resistant PrP^Sc^ are defined (Types 1, 2, and 3). In combination with the codon 129 genotype, six subtypes of sCJD are commonly observed: 1MM, 2MM, 2MV, 2VV, 3MV, and 3VV. (**D**) PrP^Sc^ pathology in the frontal cortex from an sCJD patient with the MM1 subtype showing diffuse deposits in a punctate or “synaptic” staining pattern. (**E**) A PrP^Sc^ plaque in the frontal cortex from an sCJD patient with the MV2 subtype.

sCJD can be classified by immunoblot, using the molecular masses of the PK-resistant PrP^Sc^ fragments and the genotype at codon 129 of the *PRNP* gene, where either a methionine or valine residue can be present^32,52,57,58^. In the most commonly used classification system, six subtypes of sCJD have been defined using these biochemical and genetic features. There are two possible sizes of the unglycosylated PK-resistant PrP^Sc^ fragment: “Type 1” PrP^Sc^ has a molecular mass of ~21 kDa whereas “Type 2” PrP^Sc^ has a mass of ~19 kDa. In conjunction with the three possible genotypes at codon 129 (MM, MV, or VV) (**Figure 3** [A]), there are 6 possible combinations^32^ (**Figure 3** [B]). These subtypes are referred to as MM1, MM2, MV1, MV2, VV1, and VV2. An alternative classification defines three distinct immunoblot profiles (Types 1-3) and also results in six main subtypes (1MM, 2MM, 2MV, 2VV, 3MV, 3VV) (**Figure 3** [C])^58^. MM1 is the most common sCJD subtype in both classification systems. However, based on different molecular masses of the PK-resistant PrP^Sc^ fragments and clinical characteristics within the subtype, one team of investigators subdivided MM1 into two groups^58^. It was later argued that this division is artificial and that heterogeneity within the MM1 group can, at least in part, be explained by differences in sample preparation techniques^59^. Complicating matters, it has also been revealed that different PrP^Sc^ strain types can coexist within the brain^60-62^.

sCJD patients with MM1 or MV1 PrP^Sc^ in their brains typically present with either the classic sCJD clinical phenotype or the Heidenhain variant. Patients with MM2, MV2, or VV2, on the other hand, have a clinical phenotype characterized by dementia and ataxia. VV1 is the least common sCJD subtype and usually presents in relatively young patients^63^. MM2 sCJD has been further subdivided into MM2-cortical (MM2C) and MM2-thalamic (MM2T) based on where the predominant neuropathological changes are found within the brain. Patients with MM2T (also known as sporadic fatal insomnia) tend to present at a young age with a variety of neurological symptoms, including mental disturbances and progressive insomnia^64^. Thus, MM2T may represent the spontaneous equivalent of FFI. These phenotypic differences between individuals carrying identical genotypes could be explained by the presence of unique prion strains.

Transmission of human prion disease cases to wild-type mice or transgenic mice expressing human PrP has been a useful method for discriminating and classifying prion strains^65-67^. For example, this technique was used to prove that vCJD and BSE are caused by the same prion strain^36^. Interestingly, the bank vole, which is highly susceptible to CJD prions, has emerged as a powerful bioassay paradigm for delineating CJD strains^68^. To define the transmission properties of the sCJD subtypes, brain homogenates from each of the six subtypes of sCJD (MM1, MV1, VV1, MM2, MV2, VV2) were injected into knock-in mice expressing human PrP with different polymorphisms at codon 129^69^. While all six subtypes were transmissible, differences in incubation periods, resultant PrP^Sc^ immunoblot profiles, and vacuolation patterns in the brain provided evidence for the existence of four distinct sCJD strains: MM1/MV1, MV2/VV2, MM2, and VV1.

### 3.2 Histopathological Correlates of sCJD Subtypes and Other Prion Diseases

The MM1 and MV1 sCJD subtypes both show homogeneously distributed pathology within the entorhinal cortex, occipital cortex, and cerebellum of afflicted individuals. No significant differences regarding the pathological features or the immunoblot profile were found between MM1 and MV1 subtypes^52^. In brains of sCJD patients with subtypes MM2, MV2, and VV2, gray matter nuclei, including the thalamus, are more affected compared to MM1 and MV1. Plaques can be detected in the Purkinje cell layer of the cerebellum, especially in specimens of patients with ataxia^32^. The uncommon VV1 subtype showed prominent hippocampal pathology with the thalamus and cerebellum being less affected^32^.

Different types of human prion diseases exhibit different features following IHC for misfolded PrP. For example, both MM1 and MV1 sCJD subtypes display a PrP^Sc^ pattern that has been termed “punctate” or “synaptic”^32^ (**Figure 3** [D]). While PrP^Sc^-containing amyloid plaques have been found in GSS patients, not all plaques in prion diseases are composed of PrP^Sc^ amyloid. For instance, in the VV2 sCJD subtype, focal PrP^Sc^ aggregates that look like plaques can be negative for Congo red^32^. The MV2 sCJD subtype most commonly presents with Kuru-like PrP^Sc^ plaques (**Figure 3** [E]), whereas MM2C patients exhibit perivacuolar PrP^Sc^ deposits. Interestingly, MV2 sCJD has recently been divided into two groups: MV2K, which presents with the typical Kuru-like plaques, and MV2C, which exhibits an MM2-like PrP^Sc^ distribution pattern^70^. Heterogeneity in PrP^Sc^ staining patterns within sCJD subtypes is also possible. For example, in a recent publication on MM2T sCJD, one case showed scant PrP^Sc^ immunoreactivity in both the cortex and thalamus, while the other case showed a synaptic PrP^Sc^ pattern with no detectable deposits in the thalamus^64^. A plausible explanation for this heterogeneity would be the existence of distinct prion strains resulting in a variety of histochemical PrP^Sc^ staining patterns.

## 4. The Structural Biology of Prions and Prion Strains

The high-resolution structure of PrP^Sc^ remains a mystery. Several current structural models exist for PrP^Sc^, including the β-helix (also known as a β-solenoid) model^71^, the β-spiral model^72^, and various permutations of a parallel in-register β-sheet model^73-75^. It is challenging to determine which of the current models, if any, best represent the authentic structure of brain-derived PrP^Sc^, as they all incorporate different facets of data from low-resolution experiments. Elucidating the structure of PrP^Sc^ will be integral for understanding strain differences in prion diseases, deciphering the mechanism of prion replication, and for the development of therapeutics. PrP^Sc^ possesses many characteristics that create challenges when trying to solve its three-dimensional structure. Some of these include the high molecular weight, insolubility, and hydrophobicity of PrP^Sc ^aggregates, which hinders classical structural determination techniques such as nuclear magnetic resonance (NMR) spectroscopy or x-ray crystallography.

### 4.1 Approaches in Structural Studies: Synthetic Prions

Due to the difficulties in the isolation of PrP^Sc^ from diseased animals for structural studies, considerable effort has been put into developing synthetic prions that mimic bona fide PrP^Sc^ with respect to its biological, biochemical and transmission properties. The first successful generation of synthetic prions was achieved in 2004 and was based upon refolding of recombinant mouse PrP into amyloid fibrils^76^. After long incubation periods, these fibrils produced prion disease when inoculated into transgenic mice. In recent years, other methods for generating synthetic prions from recombinant PrP have been described^77-82^. An alternative approach to the generation of synthetic prions is to use PrP^C^ purified from mammalian cells as a substrate for PrP^Sc^-templated *in vitro* prion replication. This strategy has resulted in the generation of prions with levels of infectivity that approach those of brain-derived PrP^Sc^83-85^^.

There is still much progress that remains to be made on synthetic prions^86^. For instance, most synthetic prion preparations exhibit miniscule levels of infectivity compared to brain-derived PrP^Sc^. X-ray diffraction data show that there are substantial structural differences between amyloids produced from recombinant PrP and authentic brain-derived prions^87^. Possible explanations for the the lack of infectivity in synthetic prions is that only a small fraction of PrP molecules possess a similar structure to brain-derived prions amongst a large background of non-infectious conformers^87^, or that synthetic prions constitute a distinct strain that is less pathogenic than brain-derived prions.

### 4.2 Approaches in Structural Studies: Low-Resolution Techniques

Initial findings using classical circular dichroism and Fourier-transform infrared (FTIR) spectroscopic techniques have found that PrP^Sc^ isolated from diseased brains was mostly comprised of β-sheets instead of α-helices, which was consistent across many different strains^88,89^. Furthermore, strain-specific differences in these β-sheet secondary structures were apparent^89^. Both transmission electron microscopy (TEM) and atomic force microscopy (AFM) have been utilized to examine the ultrastructural features of prions^90^. Strain-dependent characteristics such as width, periodicities and spiral directionalities of the protofilaments have been found using these methods. X-ray fiber diffraction is also often used to generate low-resolution diffraction patterns, as PrP^Sc^ isolates do not form adequate crystals for use in X-ray crystallography. This technique has been used to provide evidence that PrP^Sc^ fibrils contain a β-helix structural motif^87^.

Recent studies utilizing hydrogen-deuterium (H/D) exchange coupled to either mass spectrometry (HXMS) or NMR spectroscopy (HXNMR) have begun to provide strain-specific structural information on specific residues within PrP^Sc^. Distinct strains of prions isolated from sCJD subtypes (MM1 and MM2) differ greatly with regards to their structural organization^91^. Backbone amide H/D exchange coupled with mass spectrometry, and histidine H/D exchange mass spectrometry data show differences at both the secondary structural level within the polypeptide backbone as well as in the quaternary packing arrangements within their β-sheets^91^. In autocatalytic recombinant PrP^Sc^ strains that are similar in origin and biochemical behaviour, HXMS data suggests that specific structural features that allow for accommodation of specific post-translationally modified PrP^C^ molecules play a crucial role in PrP^Sc^ infectivity^92^. Taken together, the H/D exchange technique offers many avenues for probing strain-dependent PrP^Sc ^structures. However, continual development of high-resolution methods for analyzing prion structure will still be required. Techniques such as cryo-electron microscopy, a form of TEM that permits examination of PrP^Sc^ structure in its native environment, without the need for crystallization, have already started to become integrated into more recent publications and show great promise for the future^93^.

### 4.3 The Structural Biology of Yeast Prion Strains

Yeast prions are self-propagating protein aggregates that play diverse functional roles in *Saccharomyces cerevisiae*^94,95^. Examples of yeast prions include [URE3] and [PSI+], with the capital letters and brackets denoting dominant, cytoplasmic inheritance of non-genetic traits. [PSI+] and [URE3] are encoded by the Sup35 and the Ure2 proteins, respectively. Like mammalian prions, yeast prion aggregates can exist as distinct strains, and strains of both the Sup35 and Ure2 proteins have been described^96,97^.

The Sup35 protein structure contains 3 domains: an unstructured C-terminal domain that contains binding sites for interaction partners such as Sup45, a highly charged middle (M) domain, and the N-terminal (N) domain, also known as the prion domain, which contains an asparagine/glutamine-rich region. Normal, soluble Sup35 forms a translational termination complex with Sup45 to halt translation at the proper Stop codon^98^. In [PSI+] cells, Sup35 is present in an aggregated state, leading to occasional read-through of Stop codons. [PSI+] propagation requires the N domain, and overexpression of only the N domain can induce the formation of long amyloid fibrils characteristic of [PSI+] in wild-type yeast^99,100^. Furthermore, aggregates formed from the same N domain sequence can give rise to [PSI+] fibrils with different biochemical properties (e.g. differential infectivity, differential resistance to protease digestion, etc.)^100^. Hence, strain variations in [PSI+] are likely due to differences in prion conformation rather than changes in the peptide sequence^100^.

Although the issue of whether yeast prions are beneficial or not to the host cell is extremely controversial^101,102^, these studies still provide support for conformation-dependent strain variability. While [PSI+] prions in some colonies have been shown to enhance growth rate under stressful conditions (e.g. in the presence of elevated ethanol concentration, inhibitors of DNA replication, pH changes, etc.), [PSI+] prions in other colonies have had neutral, toxic, or even lethal effects on cell growth^103,104^. Since [PSI+] was induced by overexpression plasmids that bear the same Sup35 sequence, variations in the phenotypic consequence of [PSI+] is again likely attributed to conformational diversity, suggesting that conformation influences the evolutionary uses of a strain.

The biggest contribution yeast prions have made to our understanding of mammalian prion biology is the notion that precise structural differences in protein aggregates underlie the phenotypic differences observed amongst strains^105,106^. Sup35NM, which consists of the N and M domains of Sup35, was used to form two [PSI+] strains at 4 °C and 37 °C that were termed Sc4 and Sc37, respectively^105^. Using HXNMR, researchers elucidated a highly protected core amyloid region that consists almost entirely of glutamine and asparagine residues within the first 40 residues of the N domain in both Sc4 and Sc37^107^. However, the amyloid core of Sc37 extends to residue 70^107^, which likely increases fibril stability and interferes with chaperone-mediated replication, offering a possible explanation of why it has lower infectivity than Sc4^105^. Supported by differences in the amyloid core region, this study helped solidify the hypothesis of conformation-dependent strain variance.

## 5. Prion strain transmission, adaptation, and mutation

Prolonged incubation times and/or low levels of infectivity often characterize the transmission of prion strains from one species to another. This phenomenon can be classified into two types of barriers: species and strain. A species barrier is largely determined by the primary structure of PrP, which requires sequence homology between the infecting PrP^Sc^ species and host-expressed PrP^C^108^^. Strain barriers are governed by structural and conformational preferences amongst PrP^Sc^ and PrP^C^, independent of primary structure^109^. Oftentimes, species and strain barriers are grouped together and referred to as transmission barriers^110^. Interestingly, bank voles (*Myodes glareolus*) appear to lack an appreciable species barrier since they are susceptible to prion strains from many different species^68,111,112^, and bank vole PrP has been touted as a “universal acceptor” for prions^45,113^.

### 5.1 Species and strain barriers

An early explanation for the existence of species barriers came from research in which it was determined that PrP^Sc^ served as a template to convert homologous PrP^C^ into PrP^Sc^108^^. Using transgenic mice expressing hamster PrP^C^ researchers were able to successfully induce hamster PrP^Sc^ formation when the mice were inoculated with hamster prions. However, this inoculum was unable to induce mouse prions. Conversely, inoculation with mouse prions induced the formation of mouse PrP^Sc^, but did not generate any hamster PrP^Sc^108^^. This demonstrated the existence of a species barrier based on homology between the source of the prion inoculum and the endogenous prion protein. This is further supported by studies using transgenic mice expressing a mouse/human chimeric PrP (MHu2M), which differed from mouse PrP at 9 positions between codons 96-167^114^. The MHu2M chimeric mice were susceptible to inoculation with human prions indicating that homology in the central region of PrP is necessary for p ropagation of human strains. However, mice expressing full-length human PrP (HuPrP) were not susceptible to inoculation with human PrP^114^. A subsequent study then demonstrated that ablation of endogenous mouse PrP in the HuPrP expressing mice was sufficient to permit infection with human prions^115^.

As research in prion disease progressed it became increasingly evident that the species barrier was not entirely sufficient to explain differences in prion strain transmission efficiency. As such, the concept of strain barriers was put forward to explain conformational requirements during prion replication. The most predominant example of strain barriers is the difference between sCJD and vCJD prions, which involve the same PrP primary sequence, yet exhibit different transmissibility in the same host^110^. vCJD is readily transmitted to non-transgenic mice, but not to transgenic mice expressing human PrP. The opposite is true for sCJD prions. While these strains share a primary structure, they differ in their glycan pattern, which likely contributes to the strain barrier^36,116^.

Despite the presence of species barriers, interspecies prion transmission is possible, typically with long initial incubation times. Often, when a substantial transmission barrier is present, interspecies transmission of prions leads to the emergence of new prion strains^117^. For example, while BSE prions can be fairly easily transmitted to wild-type mice^36,116^, the resultant mouse strain bears little resemblance to the original BSE strain in terms of its relative susceptibility to inactivation^118^. Furthermore, as previously mentioned, transmission of TME prions into hamsters produces two distinct strains: HY and DY^24^. Finally, transmission of the Sc237 strain of hamster prions to transgenic mice expressing a chimeric mouse/hamster prion protein led to the production of a prion strain that was conformationally distinct from the original Sc237 prions^119^. It is important to note that not all instances of interspecies prion transmission lead to the emergence of novel strains^120^. The mechanism of prion strain mutation upon interspecies prion transmission is thought to involve selecting a minority of PrP^Sc^ conformers, which are either present at low levels in the original strain or arise due to conformational mutation, that are better suited to the conformational preferences of PrP^C^ expressed in the new host^121^.

### 5.2 Prion Strain Mutation and Evolution

**Figure 4:**
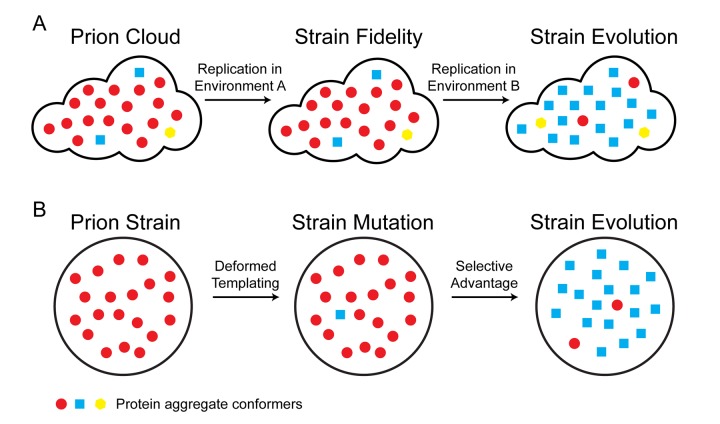
The cloud and deformed templating hypotheses for prion strain evolution (**A**) In the cloud hypothesis, there is pre-existing conformational heterogeneity within a prion strain. A single dominant sub-strain (red circles) is responsible for the bulk properties of the strain in a given environment. If the strain is shifted to a new environment (such as the presence of an anti-prion drug), a minor sub-strain (blue squares) that is more suited to the new conditions may emerge as the new dominant conformer, resulting in the “evolution” of the prion strain. (**B**) In the deformed templating hypothesis, the templated replication of a single conformational state (red circles) may occasionally be imperfect, leading to production of a conformationally distinct molecule (blue square). If this new isoform has a selective advantage in the current replication environment, it may eventually take over and become the dominant conformer.

The prion replication process may not always be fully faithful as subsequent generations of prions may not be structurally identical to the parental PrP^Sc^ strain. Instead, a heterogeneous mixture of new and old prion conformers may be formed, creating a quasi-species^122^. This ensemble of structures has been referred to as the prion “cloud”. The cloud hypothesis posits that prion strains are not clonal and are instead intrinsically heterogeneous, consisting of major and minor PrP^Sc^ variants (**Figure 4** [A])^121^. The existence of prion clouds offers a potential explanation for several phenomena that have been observed during prion replication, including apparent prion strain selection and/or mutation as well as the acquisition of drug resistance in prions.

Research on prion proteins in the lab has shown that the environment in which the prion is situated plays a role in its evolution and mutation. For example, if a prion cloud that has a dominant strain is exposed to an altered environment it can result in increased propagation of a minor strain. If this strain is run through multiple replication cycles in the same environment it will now become the dominant prion strain within the cloud. This indicates that prion proteins evolve through natural selection^123^, meaning that the conformer that is most suited for a particular environment will thrive and become the dominant strain. Conversely, strains that are not suited to a particular environment will not thrive and may eventually be eliminated from the cloud with enough prion replication cycles^124^.

Strong evidence for the existence of prion clouds has come from studies on prion-infected cultured cells. Infection of cells with a supposedly cloned prion strain resulted in the gradual diversification of the strain away from its original properties^123^. Moreover, treatment of cells with swainsonine, an inhibitor of protein glycosylation, resulted in the selection of a prion strain that was resistant to the drug; when the drug was removed, the original drug-sensitive strain re-emerged as the dominant species^123^. Propagation of prions in the presence of an inhibitory drug may not only cause selection of drug-resistant prions but also of stable variants that propagate more efficiently in the presence of the drug^125^. Repeated passaging of synthetic prion strains in cultured cells also resulted in an eventual shift in prion strain properties^126^.These results show that prions are susceptible to Darwinian evolution, at least in cultured cells. Prion strain selection has also been observed in transgenic mice expressing chimeric mouse/human PrP inoculated with vCJD prions. Mice with longer incubation periods exhibited a phenotype consistent with the original vCJD strain, whereas mice with shorter incubation periods exhibited a more sCJD-like phenotype^127^. The existence of a cloud of prion conformers in the original vCJD isolate could explain these results.

Another mechanism proposed to explain prion strain heterogeneity is the deformed templating hypothesis (**Figure 4** [B]). This mechanism was proposed in order to explain the behaviour of synthetic prions upon propagation in hamsters^128-131^. Initial passage of synthetic prions produced atypical species of PK-resistant PrP that were pathologically silent. Upon repeated passaging, stereotypical PK-resistant PrP^Sc^ emerged, resulting in the canonical biochemical and pathological markers of prion disease. The deformed templating hypothesis speculates that the original structure of synthetic prions is distinct from typical PrP^Sc^, but is still able to self-replicate. However, an imperfect templating process results in the occasional generation of altered PrP conformers, some of which mimic authentic PrP^Sc^, which eventually gain a selective advantage due to their increased pathogenicity and thus become the dominant species. This theory is more in line with Lamarckian evolution rather than Darwinian evolution^124^, but it is believed that these mechanisms are not mutually exclusive and most likely both play a role in prion mutation and evolution.

The acquisition of drug-resistance in prions following prolonged treatment with potential anti-prion therapeutics is an active area of research. The concept of drug resistance is more commonly applied to bacteria and viruses that can acquire resistance via changes in their nucleic acid genomes. With prions, the mutations likely occur at the level of protein aggregate structure, resulting in the generation of conformationally distinct prion strains. The notion of drug-resistance acquisition appears to apply to yeast prion strains as well^132^. Drug-resistance following treatment with an anti-prion compound was first observed with the drug quinacrine, which can effectively cure dividing cells infected with mouse, but not CWD prions^133,134^. However, while treatment of prion-infected mice or non-dividing cells with quinacrine resulted in an initial drop in PrP^Sc^ levels, this reduction was only transient and PrP^Sc ^levels inevitably began to rise again^135^. The biochemical properties of the prions obtained post-quinacrine treatment were different from the original strain, suggesting that quinacrine was able to eliminate specific prion sub-strains, resulting in the emergence and selection of a drug-resistant variant.

Chronic treatment of mice with 2-aminothiazoles, another class of anti-prion molecules, also leads to the emergence of drug-resistant prion strains^136^. However, unlike quinacrine, the 2-aminothiazoles were able to extend the lifespan of prion-infected animals, suggesting that they are more effective at clearing prions, although the end result was the same. This study also revealed that anti-prion drugs are strain-specific: the 2-aminothiazoles had no effect on the replication of human prion strains^136^. More recent research has shown that the generation of drug-resistant prion strains is not an inevitable consequence of exposure to anti-prion therapeutics^137^. Although aryl amide compounds were able to extend the lifespan of prion-infected mice, the resultant prions were not drug-resistant, at least upon propagation in cultured cells. These studies have provided evidence that intermittent therapy with a mixture of anti-prion compounds may be required to slow or stop the progression of prion disease and prevent the emergence of drug-resistant strains.

## 6. Strains of Protein Aggregates in Other Neurodegenerative Diseases

Over the past 10 years, considerable evidence has been obtained suggesting that the pathological protein aggregates characteristic of common neurodegenerative disorders, such as Alzheimer’s disease (AD) and Parkinson’s disease (PD), exhibit properties reminiscent of prions. Most notably, these aggregates have been shown to be capable of self-propagation, allowing them to spread from cell-to-cell within the brain in a prion-like fashion^138-140^. Evidence for this prion-like behaviour has come from neuropathological analysis of human brain tissue, which has revealed an ordered, stereotypical progression of protein aggregation in the CNS as well as evidence for cell-to-cell transfer of protein aggregates^141-144^; the observed propagation of protein aggregates between interconnected regions of the mouse brain^145,146^; prion-like “transmission” studies in which pre-formed protein aggregates seed the formation and spread of aggregates in the brains of mice^147-152^; and experiments that demonstrate the uptake and cell-to-cell transfer of protein aggregates^153-159^. The prion-like propagation hypothesis for neurodegenerative diseases remains controversial^160^ and the debate on whether non-PrP self-propagating protein aggregates should be referred to as “prions” or something else (prionoids, prion-like, etc.) is ongoing^161-164^. In recent years, evidence for the existence of distinct strains of non-PrP neurodegenerative disease-associated protein aggregates has emerged, further strengthening the notion that these aggregates exhibit prion-like properties.

### 6.1 Strains of Aβ Aggregates

AD is a slowly progressive dementia that is pathologically defined by the presence of intracellular neurofibrillary tangles composed of tau protein, and extracellular amyloid plaques comprised of fibrillar β-amyloid (Aβ) peptide. The predominant components of Aβ deposits are Aβ40 and Aβ42peptides, which are generated by the proteolytic cleavage of the amyloid precursor protein (APP) by β- and γ-secretases. There is considerable evidence that Aβ aggregates, either isolated from diseased brain tissue or prepared from synthetic peptides, are capable of exhibiting prion-like self-propagation upon injection into susceptible transgenic mice^165^.

Amyloids are unbranched protein fibrils consisting of repeating β-strands perpendicular to the fibril axis, forming a cross-β-sheet, with hydrogen bonds running parallel to the axis^166-168^. The formation of structurally distinct polymorphs, or strains, is now recognized as a common property of amyloid fibrils^167,168^. Four models have been proposed to explain the molecular basis of amyloid strains^167,169^. Packing polymorphs have the same residues in the cross-β core but vary in terms of parallel or anti-parallel strand packing. Segmental polymorphs differ in the residue segments that are involved in forming the cross-β core structure. Side chain polymorphism occurs when an amino acid side chain may be rotated to favour a specific orientation, thus altering the surface features. Finally, structural differences can arise due to an independent form of polymorphism within the bundles of protofilaments comprising the amyloid fibrils, known as assembly polymorphism. For Aβ, most of these models have been generated using smaller fragments of the peptide^170^.

Evidence supporting the strain-like behaviour of fibrils composed of full-length Aβ has come from studies using synthetic and brain-derived aggregates (**Table 1**). Early *in vitro *studies on synthetic Aβ40 fibrils revealed that at least two distinct strains could be generated, whose different morphologies could be controlled by subtle changes in growth conditions^171^. Aβ40 fibrils grown under quiescent conditions exhibited a predominantly “twisted” morphology, whereas agitation resulted in a mainly “striated ribbon” morphology with no resolvable twist. Pronounced differences between quiescent and agitated Aβ40 fibrils were also visible as variations in the ^13^C NMR chemical shifts, indicative of differences in the local structural and conformational environment. These strain-specific characteristics were maintained in serial seeding reactions, confirming the existence of self-propagating, molecular-level polymorphisms in synthetic Aβ40 fibrils.

Subsequent research revealed that synthetic Aβ40peptides can form a range of different aggregate morphologies, even when generated under the same conditions, and that polymorphic fibrils can exist within a sample^172^. The structural characteristics of twelve different Aβ40 fibrils grown under identical conditions were investigated, and it was concluded that synthetic Aβ40 strains share a common cross-β motif, with high structural diversity in terms of intra- and inter-residue interactions^172^. Eleven of the twelve fibril preparations exhibited two-fold symmetry whereas the twelfth fibril differed substantially, consisting of two protofilaments that were offset with respect to the central axis^172^. Strains of synthetic Aβ40 fibrils that exhibit three-fold symmetry have also been observed^173^. Another group explored eight different fibril growth conditions, producing five distinct self-propagating polymorphs of synthetic Aβ40, along with a non-fibrillar, β-sheet-rich strain that aggregated in the presence of Zn^2+^174^^. Analysis using HXMS revealed that the fibrils all exhibited different degrees of protection, indicative of major conformational differences in these strains. Given that only eight growth conditions were tested, it is highly likely that additional Aβ fibril strains can be formed.

Strain-like behaviour has also been observed in Aβ fibrils composed of synthetic Aβ42 peptide. APP23 transgenic mice inoculated with Aβ42fibrils exhibited significantly more but smaller amyloid plaques than mice injected with Aβ40 fibrils^175^, indicating that different Aβ isoforms can elicit distinct pathological phenotypes. These variances were eliminated when the synthetic Aβ40 and Aβ42 fibrils were prepared in the presence of 0.1% SDS. Thus, two preparations of Aβ42 aggregates (with or without 0.1% SDS) possessed diverse physical and biological properties, supporting the existence of at least two distinct strains of synthetic Aβ42 fibrils^175^.

Strain-like properties of Aβ fibrils have also been described for aggregates present in brain tissue. Injection of APP23 transgenic mice with brain extracts from aged APP23 mice produced a distinct pattern of induced Aβ deposition compared to APP23 mice injected with brain extracts from aged APPPS1 transgenic mice, suggesting the existence of polymorphic Aβ strains with varying biological activities^147^. A follow-up study revealed conformational differences in the Aβ aggregates present in aged APP23 and APPPS1 mice and that the conformational properties of the induced Aβ aggregates in APP23 mice inoculated with either APP23 or APPPS1 brain extract closely matched those of the injected aggregates^176^. To examine whether distinct strains of Aβ aggregates can be distinguished in the brains of AD patients, APP23 mice were**inoculated with brain homogenates from sporadic AD or familial AD cases with either the Arctic or Swedish mutations in APP^177^. The brain homogenate from the Arctic AD sample induced a distinct pattern of cerebrovascular Aβ deposition in the mice that was distinguishable from both the Swedish and sporadic AD patients, and this unique pattern was maintained upon serial passage. Since the Aβ produced in APP23 mice does not contain the Arctic mutation, these findings suggest that the conformation of Aβ aggregates determines the properties of Aβ strains rather than the specific mutation itself.

It was first reported that Aβ fibrils formed using synthetic Aβ40, Aβ42, or a mixture of both were unable to induce detectable Aβdeposition in APP23 mice^147^. More recently, induction of cerebral Aβ deposition with synthetic Aβ fibrils has been achieved, although the biological activity of synthetic aggregates appears to be considerably lower than brain-derived aggregates^148^. One possible interpretation is that synthetic and brain-derived Aβ fibrils comprise structurally distinct strains of Aβ aggregates, similar to what has been described for prion strains composed of PrP^87^. In support of this idea, one group seeded the growth of synthetic Aβ40 fibrils with brain-derived Aβ aggregates from an AD brain to investigate the molecular structure of AD-specific Aβ aggregates^178^. Solid-state NMR (ssNMR) characterization revealed that AD-seeded Aβ40 fibrils exhibited sets of chemical shifts markedly different from those of spontaneously generated synthetic Aβ40 fibrils, suggesting that conformational differences exist between brain-derived and *in vitro*-generated Aβ aggregates.

The first detailed, experimentally determined structures of brain-derived Aβ aggregates from AD patients have provided additional evidence for the existence of Aβ strains^179,180^. Using brain extract from two AD patients with distinct clinical histories to seed the polymerization of synthetic Aβ40, it was revealed that each patient possessed a single predominant, but unique Aβ aggregate structure^179^, suggesting that in AD patients a single nucleation site in the brain may give rise to a homogenous population of self-propagating aggregates. Further structural studies on seeded Aβ40 fibrils from a larger number of AD cases, including atypical variants, revealed that a single predominant Aβ40 strain is found among stereotypical, slowly progressive AD cases whereas additional structures can be found in a more rapidly progressive variant of the disease^180^. Different Aβ42conformers could also be biochemically detected in rapidly progressive AD brains compared to those in slowly progressive AD^181^. Therefore, the existence of distinct Aβ strains may, at least in part, explain the variable rates of disease progression observed in AD patients. Recent studies using X-ray microdiffraction of histological sections of brain tissue from three AD patients also showed that patients with different clinical histories contain different Aβstructures^182^. However, in two cases, distinct ensembles of amyloid structures were found to co-exist within a single tissue section. This evidence could simply reflect different stages of plaque maturation, but may also provide evidence for the presence of multiple strains of Aβ aggregates within a single brain. Additional studies using a larger number of AD samples are necessary to resolve this issue.

The distinct strains of Aβ fibrils detected *in vitro, *and particularly in AD patients, may underlie the heterogeneity in the rate of progression, pathogenicity, neuropathological presentation and clinical outcomes observed in AD. It has been suggested that certain Aβ strains might even preferentially promote neurofibrillary tangle development^183^. Further investigation into understanding and classifying the specific structural differences between and within synthetic and brain-derived Aβ strains offers great hope for developing precise and informative molecular diagnostics and therapeutic compounds for AD.

**Table 1:** Comparison of major studies examining the strain-like behaviour of Aβ aggregates

Citation	Aβ Fibril Composition	Methods	Main Finding(s)
Petkova et al. (2005)^171^	Synthetic Aβ40	TEM, ssNMR,	Different fibril morphologies contain different underlying molecular structures that can be regulated by variations in growth conditions.
Meyer-Luehmann et al. (2006)^147^	Brain-derived Aβ aggregates and synthetic Aβ40/Aβ42	IHC, immunoblotting	Phenotype of exogenously induced amyloidosis depends on both donor and recipient, suggesting existence of Aβ strains. Synthetic Aβ unable to induce Aβ deposition in transgenic mice, indicative of differences between synthetic and brain-derived fibrils.
Meinhardt et al. (2008)^172^	Synthetic Aβ40	TEM, cryo-EM, 3D reconstruction	Twelve distinct Aβ fibril morphologies can be formed under the same growth conditions, demonstrating that polymorphism exists within the same sample.
Paravastu et al. (2009)^178^	Synthetic Aβ40 seeded with AD brain extract	TEM, ssNMR	Brain-seeded Aβ40 samples have one principal structure, which is distinct from purely synthetic Aβ40 fibrils.
Kodali et al. (2010)^174^	Synthetic Aβ40	TEM, FTIR, HXMS	Showed five structurally distinct self-propagating Aβ fibrils, each having their own physical properties.
Heilbronner et al. (2013)^176^	Brain-derived Aβ aggregates	IHC, immunoblotting, LCP staining	In the brain, variances in Aβ peptide length may play a role in the type of Aβ strain formed, which can be sustained through serial propagation.
Lu et al. (2013)^179^	Synthetic Aβ40 seeded with AD brain extract	TEM, ssNMR	Found the presence of a single predominant Aβ40 structure in each of two AD patients, but they were structurally distinct from each other.
Stöhr et al. (2014)^175^	Synthetic Aβ40/Aβ42	TEM, IHC, bioluminescence imaging	The composition of Aβ plaques depends on conformation of Aβ aggregates in the inoculum, demonstrating distinct synthetic Aβ strains. The presence of 0.1% SDS can cause formation of a distinct strain of Aβ42 fibrils.
Watts et al. (2014)^177^	Brain-derived Aβ aggregates	IHC, immunoblotting, bioluminescence imaging	At least two distinct strains of Aβ are present in the brains of AD patients (Arctic vs. Swedish mutation), and are maintained upon serial passaging.
Cohen et al. (2015)^181^	Brain-derived Aβ aggregates	CDI and GdnHCl stability assay	There are a wide range of Aβ42 structures in AD cases with three distinct strain groups. Aβ42 is more heterogeneous in rapidly progressive AD than slowly progressive AD.
Liu et al. (2016)^182^	Brain-derived Aβ aggregates	X-ray microdiffraction	Brain tissue from AD patients with different clinical histories may contain different Aβ fibrillar structures, and distinct amyloid structures can coexist within a single tissue sample.
Qiang et al. (2016)^180^	Synthetic Aβ40/Aβ42 seeded with AD brain extract	TEM, ssNMR	A single Aβ40 fibril structure predominates in normal AD cases. Rapidly progressive AD cases exhibit more variable Aβ40 fibril structures. Structural heterogeneity was observed with most Aβ42 fibrils.

### 6.2 Strains of Tau Aggregates

Tau is a microtubule-associated protein that is highly soluble and disordered in its native form, and is predominantly expressed within neuronal cells of the CNS^184^. Due to alternative splicing, six isoforms of tau exist which can be divided into two major groups, 4R and 3R, based on the number of microtubule binding repeats present^185^. During disease, tau can polymerize to form insoluble, hyperphosphorylated aggregates, such as the neurofibrillary tangles found in AD patients^186^. Tau aggregation is implicated in many neurodegenerative diseases, collectively known as tauopathies, which include AD, progressive supranuclear palsy (PSP), and corticobasal degeneration (CBD), to name a few^187^. Tauopathies can be classified based on the tau isoforms that comprise the aggregates, which present with different cellular localization patterns, tau pathology and clinical symptoms^187^. This has raised the possibility of the existence of conformationally distinct tau fibril strains, which may contribute to the array of clinical symptoms associated with tauopathies.

Several studies have demonstrated that synthetic tau filaments (generated from recombinant tau) can be either homogeneous, comprised of either 3R or 4R tau isoforms, or heterogeneous, containing both tau isoforms, giving rise to four conformationally distinct tau filaments^188-190^. It was revealed that homogeneous 3R and 4R tau filaments have different seeding properties with the latter unable to seed 3R tau, thereby exhibiting a cross-seeding barrier^189,190^. Interestingly, when 3R seeds were used to generate filaments from 4R monomers, the resulting 4R filaments were able to subsequently seed 3R tau^189^. This finding suggests that cross-seeding between 3R and 4R can give rise to a new, conformationally distinct 4R tau filament that does not exhibit the asymmetric seeding barrier normally possessed by 4R filaments. Additionally, it has recently been shown that over time, homogeneous 4R tau fibrils undergo sporadic conformational change over multiple cycles of seeding, and that point mutations alter seeding selection, thereby giving rise to different fibril conformations^191-193^. It should be noted that these studies used truncated versions of 3R and 4R tau, which do not incorporate the variable regions that define the three members of each group. Thus, the number of conformationally distinct tau filaments identified to date may be grossly underestimated. The identification of conformationally distinct tau fibrils, which possess different seeding and physical properties, strongly supports the existence of tau fibril strains.

The studies mentioned thus far demonstrate the existence of unique tau conformers but they do not address their strain-like behaviour, particularly whether or not their conformational properties are maintained upon propagation in cells or organisms. Several *in vitro* and *in vivo* studies, using cultured cells and transgenic mice, have addressed these shortcomings^194,195^. One study demonstrated that two distinct cellular subclones, each exhibiting tau aggregates with different morphologies and biochemical characteristics, could be isolated following exposure to synthetic tau fibrils^194^. Moreover, upon serial passaging, the associated morphologies and biochemical characteristics of the two strains did not change, thus illustrating their robustness and stability in culture^194^. Inoculation of these two conformers into tau transgenic mice gave rise to unique pathologies in the hippocampus, which were consistent upon serial propagation through multiple passages in mice^194^. Most impressively, the two strains were biochemically stable when passaged from mice back into naïve cultured cells, maintaining their initial phenotypes, consistent with the behaviour of strains^194^. A recent, more detailed study analyzing 18 distinct tau conformers found similar results^195^. The putative strains gave rise to diverse pathologies and exhibited different spreading rates, thereby making it conceivable that tau strains underlie the array of clinical symptoms and progression rates observed across the tauopathies.

A limitation of these aforementioned studies is that they employed synthetic tau strains generated from truncated forms of tau, which may alter some of the observed phenotypes. In fact, it has been shown that synthetic tau fibrils possess different seeding efficiencies than brain-derived tau aggregates due to significant conformational differences^196^, and that recombinant tau seeded with AD-derived tau aggregates forms fibrils that resemble the original AD structures but not spontaneously generated recombinant tau fibrils^197^. Brain-derived tau aggregates have also been shown to exhibit strain-like behaviour. When transmitted to cultured cells, tau aggregates from the various tauopathies produce unique aggregate morphologies^194^. When brain homogenates, isolated from various patients with different sporadic tauopathies, were injected into mice, they recapitulated certain pathological features of their associated human diseases including the morphology of tau inclusions and their biochemical characteristics^198^. When transgenic mice expressing wild-type 4R tau were injected with Pick’s disease aggregates, which are predominantly comprised of 3R tau fibrils, fewer induced tau inclusions were observed than in mice injected with either PSP or CBD aggregates, which are predominantly made up of 4R tau fibrils^198^, suggesting that tau strains preferentially propagate with specific tau isoforms. Similar results have been obtained with infection experiments that utilize cells expressing either 4R or 3R tau isoforms^199^. More recently, tau aggregates extracted from AD brains, but not recombinant tau fibrils, were shown to have the ability to propagate abundant tau pathology in multiple brain regions of non-transgenic mice^200^. Interestingly, some of the brain homogenates in these studies contained multiple disease-associated tau fibrils suggesting that an ensemble or ‘‘cloud’’ of these conformers or strains exist within individuals, which could account for the range of phenotypes observed within an individual patient^198^.

Tau aggregates must meet a number of criteria in order to be classified as bona fide strains including existing as diverse conformers that can be stably propagated through living organisms, possessing variations in biochemical characteristics such as seeding efficiencies, toxicity, solubility, and aggregate size, and the ability to produce an array of pathologies, which would account for the range of symptoms underlying the tauopathies. Studies of both synthetic and brain-derived tau aggregates have provided evidence that at least certain species of tau aggregates do indeed meet these criteria, thereby reinforcing the notion that tau aggregates, like PrP aggregates, can exist as unique strains.

### 6.3 Strains of α-Synuclein Aggregates

α-Synuclein (α-Syn) is a 140-residue phospholipid-binding presynaptic protein that is abundantly expressed in the brain and exists primarily as an intrinsically disordered monomer, although there is some evidence that it can assemble into an α-helical tetramer^201-203^. Although the precise function of α-Syn is not entirely known, it seems to play a role in the regulation of synaptic vesicular release of neurotransmitters by promoting the assembly of SNARE complexes. The α-Syn protein is encoded by the *SNCA* gene, with missense mutations and multiplications of this gene linked to autosomal dominant familial forms PD. The synucleinopathies are a group of neurodegenerative disorders characterized by abnormal accumulation and deposition of α-Syn in the brain. There are three main synucleinopathies: PD, dementia with Lewy bodies (DLB), and multiple system atrophy (MSA)^204^. Both PD and DLB patients exhibit neuronal α-Syn inclusions in the form of Lewy bodies (LBs) and Lewy neurites (LNs). However, in DLB the LBs are found mainly in cortical brain areas whereas in PD patients they are initially found in subcortical structures such as the substantia nigra. The predominant pathological feature of MSA is the formation of α-Syn inclusions called glial cytoplasmic inclusions (GCIs), which are most commonly observed in oligodendrocytes. Although all three of these disorders display α-Syn pathology, the different clinical and neuropathological manifestations could suggest that distinct α-Syn strains are present.

The evidence supporting the existence of α-Syn strains has come from studies investigating both brain-derived and recombinant forms of the protein (**Table 2**). One group has investigated the impact of different assembly conditions on the formation of α-Syn fibrils^205^. Changing the salt concentration when polymerizing monomeric α-Syn led to the formation of two different types of assemblies: “fibrils”, which were cylindrical, and “ribbons”, which were flatter and more twisted. The fibrils were more resistant to PK digestion while the ribbons had a slightly higher β-sheet content, implying that the assemblies were conformationally distinct. Both assemblies were able to imprint their intrinsic structure to monom1eric α-Syn, indicating that they are self-propagating. Moreover, α-Syn fibrils were found to be more toxic than ribbons when applied to cultured cells. Collectively, these results suggest that the “fibrils” and “ribbons” constitute distinct strains of α-Syn aggregates.

In a follow-up study, the *in vivo* behaviour of α-Syn oligomers, ribbons and fibrils were assessed following injection into the rat substantia nigra in the presence or absence of recombinant adeno-associated viral vector-mediated overexpression of human α-Syn^206^. Only the fibrils and ribbons induced formation of LB- and LN-like inclusions in dopaminergic neurons. These inclusions, which contained phosphorylated α-Syn, were more abundant for the α-Syn ribbons. Overexpression of α-Syn enhanced neurodegeneration in a strain-dependent manner, with fibrils inflicting greater neurotoxicity on the striatonigral pathway while ribbon inoculation gave rise to more LB/LN-like inclusions^206^. Overall this study provided further support for the existence of α-Syn strains based on the numerous differences observed in the properties of α-Syn fibrils and ribbons.

Evidence suggesting the existence of α-Syn strains in brain-derived samples has also been obtained. A recent study revealed that inoculation of M83 transgenic mice, which express mutant human α-Syn^207^, with brain homogenates from MSA patients resulted in a significantly faster progression of neurological disease compared to the same mice inoculated with brain homogenates from aged, spontaneously ill M83 mice^208^. Mice inoculated with MSA extract developed signs of disease with an incubation period of around 100 days while mice injected with M83 extract developed neurological dysfunction with an incubation period of approximately 210 days. These incubation period differences suggest that the α-Syn aggregates found in MSA brains comprise a conformationally distinct α-Syn strain compared to the ones found in aggregates formed spontaneously in the brains of aged M83 mice^208^. A follow-up study demonstrated that the incubation period differences were maintained upon second passage, revealing that α-Syn aggregate strains, like prion strains, are serially transmissible^209^. Additionally, inoculation of M83 mice with PD brain extract did not produce neurological illness^209^, potentially indicating that PD is caused by a much slower progressing strain of α-Syn aggregates. Indeed, MSA progresses much more rapidly in patients than PD^210^.

Another study demonstrated that the distribution of self-propagating α-Syn aggregates within the brain can vary among MSA patients^156^. Using a cellular infection assay, there were significant differences in the levels of self-propagating α-Syn aggregates across four different brain regions from three MSA patient brains. Interestingly, the pathological distribution of GCIs was similar in all three patients. One possible interpretation of these results is that each of the three MSA patient samples contained a different α-Syn strain^156^. However, conformational differences between the α-Syn aggregates in the MSA cases were not investigated, and additional studies are required to confirm the presence of distinct strains.

Additional support for the existence of α-Syn strains was revealed by a study that investigated the link between α-Syn and tau pathology^211^. The repetitive self-seeding of α-Syn monomers with α-Syn fibrils formed in each previous passage led to the emergence of a new strain with distinct tau cross-seeding properties. Neurons treated with the non-seeded fibrils, called “strain A”, demonstrated a completely distinct pattern of α-Syn and tau inclusions compared to the fibrils generated via repetitive seeded fibrillization, called “strain B”. In general, strain B was much better at eliciting tau pathology. Moreover, following inoculation of strain A or B into the hippocampus of PS19 transgenic mice, which express mutant human tau^212^, strain B-injected mice displayed notably more tau inclusions in all parts of the hippocampus and locus coeruleus. Together these data illustrate the significantly enhanced tau cross-seeding ability of strain B fibrils, both *in vitro *and *in vivo.* The substantial differences in the functional properties, seeding capacity and structural characteristics of these two synthetic α-Syn conformers closely parallel the distinctions found among different prion strains.

The finding that a spectrum of α-Syn strains can manifest *in vitro* due to minor perturbations, such as repetitive seeding^211^ or changes in salt concentration^205^, gives credence to the formation of diverse α-Syn strains in an environment as biochemically complex as the human brain. This idea is also supported by the protein-chameleon concept, which states that human α-Syn is intrinsically unstructured and is able to adopt various conformations due to its structural plasticity^213^. In addition, the mechanism of interneuronal α-Syn pathology spreading may involve the same repetitive seeding that led to the emergence of strain B fibrils from strain A fibrils^211^, potentially suggesting that divergent α-Syn strains may emerge during disease progression and possibly accounting for the morphological differences observed in LBs within PD patient brains.

**Table 2:** Comparison of major studies examining the strain-like behaviour of α-Syn aggregates

Citation	α-Syn Strain	Type	Structure	Disease Phenotype	Areas Affected	Diffusion of α-Syn Within Brain	Ability to Cross-Seed Tau	Toxicity and/or Pathology	System(s) Used to Study Pathology
Bousset et al. (2013)^205^ and Peelaerts et al. (2015)^206^	Ribbons	Recombinant	Flat fibrils	Similar to PD and DLB	Deposits in CNS (after intravenous injection)	Less than α-synuclein oligomers	Not investigated	LB/LB-like inclusions	Rats expressing human α-Syn
	Fibrils	Recombinant	Cylindrical fibrils	Progressive motor impairment and cell death	Deposits in CNS (after intravenous injection)	Less than α-synuclein oligomers	Not investigated	Neurotoxic burden on the striatonigral pathway, more toxic than Ribbons	Rats expressing human α-Syn
Guo et al. (2013)^211^	Strain A	Recombinant	De novo fibrils	α-Syn pathology	Perikarya and processes of neurons	Spreads faster than Strain B	Limited	Highly toxic, increased lactate dehydrogenase release and reduced metabolic activity	Primary neurons and PS19 tau transgenic mice
	Strain B	Recombinant	Serially seeded fibrils	Tau pathology and α-Syn pathology	Cytoplasmic, but mostly in neurites	Spreads slower than Strain A	Efficient	No impact on cell survival	Primary neurons and PS19 tau transgenic mice
Watts et al. (2013)^208^ and Prusiner et al. (2015)^209^	MSA brain extract	Brain-derived	Insoluble α-Syn aggregates	Progressive CNS dysfunction with α-Syn pathology	Brainstem and midbrain (after intracerebral injection)	Shorter incubation period compared to M83 strain	Not investigated	Intraneuronal deposits of phosphorylated α-Syn	M83 α-Syn transgenic mice
	M83 brain extract	Brain-derived	Insoluble α-Syn aggregates	Progressive CNS dysfunction with α-Syn pathology	Brainstem and midbrain (after intracerebral injection)	Longer incubation period compared to MSA strain	Not investigated	Intraneuronal deposits of phosphorylated α-Syn	M83 α-Syn transgenic mice

### 6.4 Strains of SOD1 Aggregates

Amyotrophic lateral sclerosis (ALS) is a fatal neurodegenerative disease that involves degeneration of upper and lower motor neurons, with extensive variability in clinical phenotype. Currently, mutations in a number of proteins have been implicated in both familial and sporadic ALS. The first gene identified to cause familial ALS was SOD1, which encodes the superoxide dismutase 1 protein^214^. Of all the proteins potentially involved in ALS pathogenesis, the one most strongly supportive of strain-like behaviour is SOD1. The SOD1 mutations responsible for familial ALS appear to be a source of phenotypic variability, influencing disease progression^215^.

Transgenic mice that express either wild-type or mutant human SOD1 are frequently used to study the role of protein misfolding and propagation in ALS. Within these transgenic mice, two strains of SOD1 aggregates, referred to as “strain A” and “strain B”, were discovered^216,217^. Using binary epitope mapping with antibodies recognizing amino acid sequences 57-72 (exposed in strain A) or 111-127 (exposed in strain B), the presence of misfolded SOD1 could be detected in the mice before any histological signs of injury^216^. The SOD1 aggregates from mice expressing D90A-mutant protein were distinct from those in mice expressing G93A- or G85R-mutant SOD1, with the former composed primarily of strain B, while the latter of strain A^216^. Moreover, when aggregate seeds of strain A or B were injected into the lumbar spinal cord of 100-day-old G85R mice, the strains propagated faithfully since spinal cords from strain A-inoculated mice possessed strain A aggregates, while the strain B-inoculated mice possessed strain B aggregates^217^. Strain A inoculation resulted in a shorter incubation period compared to inoculation with strain B, and the two strains also differed with respect to deposition, as strain A spread along the spinal cord evenly, while strain B preferentially localized to the lumbar section^217^.

Aside from this work, spinal cord homogenates from paralysed SOD1 transgenic mice have been injected into newborn G85R-YFP and G85R-untagged mice^152,218^. Differences were seen between inocula prepared from different SOD1 mutant lines. Specifically, the G93A variant was able to seed motor neuron disease at a much faster rate than other inocula^218^. Such findings reflect human data, as the G93A mutation leads to a rapid disease progression, while other mutations used in the study are associated with slower progression. This preferential transmissibility suggests strain differences, where some conformations would be less equipped to induce misfolding and propagation^218^. In these experiments, subsequent passages of all homogenates decreased incubation time and increased the chance of disease transmission while maintaining inclusion characteristics, suggesting that host adaptation was occurring. While distinct strains of SOD1 aggregates may factor into the pathogenesis of familial ALS, there is still debate regarding the existence of wild-type SOD1 aggregates in sporadic ALS^219,220^. Interestingly, inocula composed of either homogenates from wild-type SOD1 overexpressing mice or misfolded recombinant wild-type SOD1 were able to induce paralysis with inclusion formation when given to G85R-mutant SOD1 mice^218^. The distinct pathology caused by the recombinant inoculum was preserved through multiple passages, providing further support for the strain phenomenon. However, homogenates from sporadic ALS patient samples were not able to induce inclusion pathology while those from familial ALS were, suggesting that mutant SOD1 aggregates possess certain prion-like characteristics that wild-type SOD1 from sporadic ALS cases does not^218^.

## 7. Conclusions

The existence of protein aggregate strains adds a layer of complexity to our understanding of neurodegenerative diseases and poses a considerable challenge to the development of therapeutic strategies that specifically target protein aggregation. For instance, it may be necessary to utilize human disease-relevant protein aggregate strains, both *in vitro* and in animal models, during the drug development phase to increase the chances of obtaining efficacy in patients. Moreover, strain variation, both within and between patients, may necessitate developing therapeutics that are active against a wide range of aggregate strains. Reducing levels of the normal, soluble forms of aggregation-prone proteins such as PrP^C^, tau, and α-Syn is an especially attractive strategy, as it would be predicted to be effective against all strains.

The realization that protein aggregate strains may, at least in part, explain the clinical and pathological heterogeneity in neurodegenerative diseases suggests that a greater emphasis may need to be placed on the concept of personalized medicine so that the most appropriate treatments can be administered. For example, by taking advantage of cutting-edge diagnostic techniques capable of detecting prions in biologically accessible tissues^221,222^, it may soon be possible to determine strain types in sCJD patients *ante mortem*. This may allow neurologists to better predict rates of disease progression in patients and to better classify patients prior to clinical trials.

There is now ample evidence that the concept of strains applies not only to the prion disorders, but also to more common neurodegenerative illnesses such as AD, PD, and ALS. Furthermore, evidence is mounting that other neurodegenerative disease-associated proteins, such as TDP-43 in ALS/frontotemporal dementia and huntingtin in Huntington’s disease, can also exhibit strain-like behaviour^223-225^. Even protein aggregates involved in systemic disorders, such as serum amyloid A and lysozyme, may also exist as unique strains^226-228^. With such evidence for the existence of strains in a variety of proteins that aggregate, it is worthwhile to consider the hypothesis that all proteins that misfold into disease-causing aggregates may exist in multiple misfolded states or conformations. The implications of such a hypothesis are immense for healthcare, as drug resistance could become an issue in a number of settings^109^, particularly if the aggregates exist as a cloud of conformational states. Likewise, there may be protein strains generated over time that increase the risk of transmissibility of disease across species, raising the possibility that new diseases could arise and impact human or animal health^109^. A better understanding of the drivers of the strain phenomenon and strain mutation will allow for proper preparation for the challenges ahead.

## Bullet Points

- Prion strains are distinct conformational states of prion protein aggregates that cause unique disease phenotypes in humans and animals
- Evidence is mounting that prion strains may not be structurally homogeneous, allowing strain evolution or mutation to occur when a selective pressure is present
- Protein aggregate strains may also exist in other neurodegenerative disorders, such as Alzheimer’s disease, Parkinson’s disease, and amyotrophic lateral sclerosis
